# Was the occurrence of *mycoplasma pneumoniae* pneumonia combined with cerebral watershed infarction a coincidence?: A CARE compliant case series and literature review

**DOI:** 10.1097/MD.0000000000046541

**Published:** 2025-12-12

**Authors:** Yuan-Yuan Wang, Jun-Ling Cui, Feng Mo, Ge-Fei Li

**Affiliations:** a Department of Neurology, Hebei Key Laboratory of Pediatric Epilepsy, Hebei Provincial Clinical Research Center for Child Health and Disease, Hebei Children’s Hospital, Shijiazhuang, Hebei Province, China; b Department of Neurosurgery, The Second Hospital of Hebei Medical University, Shijiazhuang, Hebei Province, China; c Department of Neurosurgery, Hebei Provincial People’s Hospital, Shijiazhuang, Hebei Province, China.

**Keywords:** cerebral watershed infarction, hypoperfusion, ischemic stroke in children, *Mycoplasma pneumoniae* pneumonia

## Abstract

**Rationale:**

*Mycoplasma pneumoniae* (MP) is one of the most common respiratory pathogens in children. The prevalence of MP-induced pneumonia (MPP) has increased significantly in China since the outbreak of pediatric respiratory diseases in 2023. MP can affect almost every organ system in the body. The incidence of cerebral infarction in MPP is relatively rare, and the mechanisms involved in the occurrence of MPP with cerebral infarction are not completely clear.

**Patient concerns:**

The clinical data of children with MPP admitted to Hebei Children’s Hospital between January 2018 and January 2024 who developed cerebral infarction during the acute stage were reviewed. Cerebral magnetic resonance imaging revealed cerebral watershed infarction in all 4 children included in the study. The thrombi were detected in other arteries in Cases 1 and case 4, and both of them tested positive for MP-PCR in their cerebrospinal fluid. Cases 2 and case 3 underwent fibrebronchoscopy under basic anesthesia and developed hemiplegia 2 hours and 1 day postoperatively, respectively.

**Diagnoses:**

Based on the clinical manifestations and laboratory test results, 4 patients could be diagnosed with MPP. These children exhibited positive neurological symptoms during the acute stage of pneumonia, considering the characteristics of the brain magnetic resonance imaging, cerebral watershed infarction can be diagnosed simultaneously.

**Interventions:**

Patient 1 and 3 received urokinase thrombolytic therapy, and was also treated with heparin sodium and aspirin for anticoagulation. Patient 2 used a combination of heparin calcium and aspirin to achieve the anticoagulant effect. Patient 4 was treated with calcium heparin for anticoagulation.

**Outcomes:**

Patient 1 and Patient 4 returned to the baseline level 1 week after the onset of the disease, while Patient 2 and Patient 3 recovered to a roughly normal state within 1 to 2 months after the onset.

**Lessons:**

Focal vasculitis, hypercoagulability and hypoperfusion may be the dominant causes of cerebral watershed infarction in patients with MP infection.

## 1. Introduction

*Mycoplasma pneumoniae* (MP) is one of the most common respiratory pathogens in children.^[1,2]^ Infants and schoolchildren are more susceptible to MP infection and constitute the majority of the susceptible population.^[3,4]^ Since the outbreak of respiratory disease in children in 2023, the prevalence of MP pneumonia (MPP) has increased significantly in China, France and other countries.^[5–8]^ Notably, MP can affect almost every organ system in the body, such as the nervous, blood, and cardiovascular systems, in addition to the respiratory system.^[9]^ For the nervous system in particular, there are already many diseases that are thought to be associated with MP infection.^[10,11]^ For examples, early-onset encephalitis/myelitis associated MP, aseptic meningitis, late-onset encephalitis/myelitis, Guillain Barre syndrome, microencephalitis and stroke.^[10,12]^ The remarkable clinical heterogeneity of it supported the existence of several pathogenic mechanisms.^[10,11]^

The mechanism of cerebral infarction during the acute stage of MPP is still unclear.^[13–16]^ Therefore, a review of children with MPP admitted to the Hebei Children’s Hospital between 2018 and 2024 was conducted in this study. A total of 4 cases experienced acute cerebral infarction during the hospitalization for MPP. Head magnetic resonance imaging (MRI) confirmed these cases as cerebral watershed infarctions, the clinical data of the 4 children who developed cerebral infarction during the acute stage were analyzed. This study was reviewed and approved by Hebei Children’s Hospital Ethics Committee, with the approval number: 202222-55 (approval date: July 4, 2022).

## 2. Case series presentation

### 2.1. Case 1

A 5-year-old boy without a history of health problems was admitted to the hospital with the chief complaint of persistent fever and dry cough for 10 days. He had received an intravenous infusion of azithromycin and ceftriaxone for 4 days before hospitalization, however, the body temperature remained > 39.0ºC. The erythrocyte sedimentation rate (ESR), C-reactive protein (CRP) level, and ferritin level were markedly elevated (Table 1). Serum titer of MP were elevated to 1:1280 on particle agglutination test, and polymerase chain reaction (PCR) revealed the presence of MP in the respiratory specimens. The results of double blood culture and sputum bacteria culture were negative. Multiple respiratory virus tests were negative for influenza, parainfluenza and other common respiratory viruses. High-resolution computed tomography (CT) of the lung revealed left inferior lobe consolidation, atelectasis, and bilateral pleural effusion.

**Table 1:** Laboratory results of 4 cases.

	Febrile days	Serum white blood cell count (×109/L)	Serum C-reactive protein (CRP) (mg/L)	Serum erythrocyte sedimentation rate (ESR) (mm/h)	Serum ferritin (μg/L)	Serum titer of MP upon admission	Serum D-dimer level upon admission (mg/L)	Serum D-dimer level at stroke (mg/L)	Serum procalcitonin level (μg/L)	Serum dehydrogenase (LDH) (U/L)	Serum cytokines detection (pg/mL)
Case 1	12	14.8	150.0	64	511.6	>1: 1280	0.94	1.28	0.663	1087	–
Case 2	10	13.0	37.39	28	464.2	1: 1280	2.78	13.77	<0.006	545	–
Case 3	18	14.1	58.54	87	–	1: 40; (2 wk later > 1: 1280)	6.09	24.68	<0.006	778	IL-6:168.72, IL-8:44.56, IL-10:21.82
Case 4	13	9.3	69.29	83	413.2	>1: 1280	3.09	6.01	0.06	588	–

Serum white blood cell count (normal reference value: 4–10 × 109/L); CRP: C-reactive protein (normal range 0–10 mg/L); serum erythrocyte sedimentation rate (ESR) (normal reference value:male 0–15mm/h, female 0–20 mm/h); serum ferritin (normal reference value:22–322 μg/l); Serum D-dimer (normal reference value: 0–0.3 mg/L); serum dehydrogenase (LDH) (normal reference value: 109–245 U/L); Serum cytokines detection, interleukin-6 (IL-6) Z(normal reference value: 0–5.30pg/ml), interleukin-8 (IL-8)(normal reference value: 0–22.60 pg/ml), interleukin-10 (IL-10)(normal reference value: 0–4.91 pg/ml).

**Figure 1.**
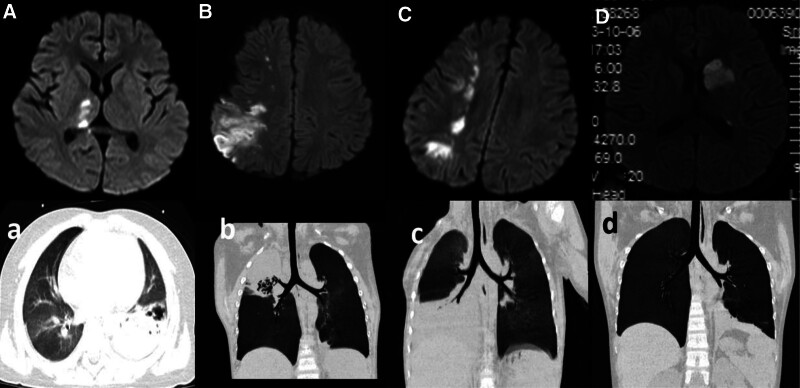
Brain MRI and chest CT findings of Cases. (A, B, C and D present images acquired during the DWI phase of patients with case 1, case 2, case 3, and case 4, respectively. a,b,c and d represent the chest CT of patients with case 1, case 2, case 3, and case 4, respectively). CT = computed tomography, DWI = diffusion weighted imaging, MRI = magnetic resonance imaging.

He became irritability, cold skin on the left side, cyanosis in the left upper limb, and decreased movement of the left upper limb with level 4 muscle strength on the third day in the hospital with azithromycin, ceftriaxone, and methylprednisolone (2 mg/kg/day). Transthoracic echocardiography revealed a strong echo band at the top of the right atrium. Doppler ultrasonography of the upper limbs revealed local widening of the distal left brachial artery and a flocculent echo, therefore, thrombosis was suspected. MRI and magnetic resonance angiography (MRA) revealed multiple abnormal intracranial signals with limited diffusion and multiple vascular walls with frizziness and uneven thickness (Fig. 1). Cerebrospinal fluid (CSF) analysis revealed a leukocyte count of 8 × 10^6^/L. The PCR of the CSF was positive for MP. The patient received thrombolytic therapy with urokinase for 3 days. Heparin sodium (100 IU/kg administered every 12 hours for 4 days), and aspirin (3 mg/kg, 2 weeks), were administered. The coagulation function was within the normal range. Muscle strength and flexibility in the limbs returned to normal after 2 days of thrombolytic therapy. The patient regained consciousness, cognitive function returned to pre-disease levels and cardiac ultrasonography revealed pericardial effusion after 1 week.

### 2.2. Case 2

A 5-year-old boy without a history of health problems was admitted to the hospital with the chief complaint of fever and cough for 4 days. Recurrent episodes of fever were recorded (4–5 times a day), and the maximum body temperature was 40.2°C. The CRP level and ESR were slightly elevated (Table 1). Particle agglutination test revealed that the serum MP antibody titer was 1:1280, and PCR revealed the presence of MP in the respiratory specimen. The results of double blood culture and sputum bacteria culture were negative. Multiple respiratory virus tests were negative for influenza, parainfluenza and other common respiratory viruses. High-resolution CT of the chest revealed a large solid shadow in the upper lobe of the right lung, high density in the lower lobe of the left lung, and a small amount of pleural effusion on the right side. Fever persisted after 5 days of treatment with azithromycin, ceftriaxone tazobactam, and methylprednisolone sodium succinate. Therefore, fibrebronchoscopy and alveolar lavage were performed. Endoscopy revealed bilateral bronchial mucosal inflammation and mucous plug blockage in the upper right branch.

Two hours after fibrebronchoscopy, the muscle strength of the left limb decreased to level 0, and the skin of the left limb was cool. MRI and MRA of the brain revealed multiple abnormal signals of different sizes in the right frontal, parietal, and occipital lobes, swelling of the right parietal cortex, and widening of the cerebral sulci and bifrontotemporal subarachnoid space. The local cerebral parenchymal arteries in the right parietal lobe decreased significantly. The posterior segment of the communicating branch of the anterior cerebral artery belonged to a multitrunk-type artery (Fig. 1). Mild leukocytosis was observed in the CSF (16 × 106/L). The PCR of the CSF was negative for MP. A combination of heparin calcium, and aspirin was administered for anticoagulation, at the same time, expansion therapy was given. Immunoglobulin (1 g/kg) was administered once daily for 2 days. The muscle strength of the left leg returned to normal after 1 month, however, the fine movements of the left hand were not flexible.

### 2.3. Case 3

A 4-year-old girl without a history of health problems was hospitalized with the chief complaint of cough and fever for 6 days. The maximum body temperature was 40.3 ºC. The symptoms did not improve after the intravenous administration of azithromycin for 3 days. The CRP level and ESR were elevated (Table 1). Chest CT scan revealed double pneumonia with partial consolidation of the right lung, small pleural effusion on the right side. PCR revealed the presence of MP in the respiratory specimens. The results of double blood culture and sputum bacteria culture were negative. Multiple respiratory virus tests were negative for influenza, parainfluenza and other common respiratory viruses. Fibrebronchoscopy and alveolar lavage were performed on the fifth and 12th days of hospitalization, respectively.

The muscle strength decreased to level 1 in the left upper limb and level 2 in the left lower limb on the 13th day of admission (1 day after the second bronchofiberscopy). MRI and MRA of the brain revealed multiple abnormal signals scattered throughout the right cerebral hemisphere with limited diffusion and widened cerebral sulci. The intracranial segment of the left internal carotid artery and the anterior segment of the left anterior cerebral artery exhibited uneven thickness, and the posterior segment of the right anterior cerebral artery and the left anterior cerebral artery were narrow (Fig. 1). The thrombin time (TT) increased to >170 seconds (normal value: 14–21 seconds) following thrombolytic therapy. The activation prothrombin time (APTT) of the enzyme increased to 64.6 seconds (normal value: 31 seconds); therefore, urine kinase treatment was discontinued. A combination of low-molecular-weight heparin sodium (100 IU/kg) and aspirin (1.5 mg/kg) was administered twice a day as anticoagulant therapy. TT and APTT were within the normal range during this period. Immunoglobulin (1 g/kg) was administered once daily for 2 days. Butylphthalein was administered to improve circulation. The muscle strength of the left limb returned to normal after 2 month.

### 2.4. Case 4

A 7-year-old girl without a history of health problems was hospitalized with the chief complaint of fever and cough for 9 days. The maximum body temperature was 38.5 ºC. The symptoms did not improve following the intravenous administration of azithromycin for 5 days. The CRP level, ESR, ferritin level, and D-dimer level were elevated (Table 1). PCR revealed the presence of MP in the respiratory specimen. The results of double blood culture and sputum bacteria culture were negative. Multiple respiratory virus tests were negative for influenza, parainfluenza and other common respiratory viruses. Chest CT scan revealed left-sided pneumonia and consolidation.

The muscle strength decreased to level 0 in the right upper limb, level 2 + in the right lower limb, and level 5- on the left side 4 days after treatment with a combination of ceftriaxone, azithromycin, and low-dose methylprednisolone sodium succinate. Arterial ultrasonography of the lower limbs revealed a solid echo in the left tibial and bilateral peroneal arteries, suggestive of thrombosis. Multiple blood pressure tests and 2 echocardiography examinations revealed no abnormality. The D-dimer level increased to 6.01 mg/L. MRI of the brain revealed abnormal signals in the left basal ganglia, left frontal parietal occipital cortex, and white matter area with limited diffusion (Fig. 1). These findings were suggestive of cerebral infarction. MRA did not reveal any abnormalities. The leukocyte count in the CSF was 5 × 10^6^/L. The PCR of the CSF was positive for MP. Heparin calcium (10 IU/kg/hour) was given as anticoagulant therapy. Butylphthalein was administered for 1 week to improve circulation. The muscle strength returned to normal.

## 3. Discussion

Correlations were observed between MP infection and the incidence of cerebral infarction at 2 time points: during the acute phase of infection and after recovery from symptomatic or asymptomatic infection.^[17,18]^ The mechanism of the former may be related to the direct invasion of MP into the central nervous system, whereas the latter may be immune-related.^[19,20]^ Hypercoagulability, destruction of vascular endothelial cells, and the promotion of coagulation by inflammatory factors may also be involved in the development of disease. However, the reported cases of cerebral watershed infarction in children are rare. This study coincidentally found 4 cases of cerebral watershed infarction during the acute phase of MP infection, which can provide reference mechanism for cerebral infarction caused by MP infection in acute stage.

Cerebral watershed infarction refers to a cerebral infarction that develops in the edge zone between adjacent areas in the brain receiving arterial blood supply. This zone is located between the large cortical artery blood supply area and the basal nucleus area between the small artery blood supply area. Cerebral watershed infarction is generally caused by haemodynamic disorders, such as systemic hypotension and hypovolaemia. Entry of microemboli into the vascular distribution area of the cerebral cortex and cerebral tissue ischemia can also cause cerebral watershed infarction.^[21]^

Case 1 and case 4 coincidentally had thrombosis in other arteries. The levels of CRP, ESR and ferritin in cases 1 and 4 were significantly higher than those in cases 2 and 3, suggesting a strong immune response. This finding supports the diagnosis of focal vasculitis. Moreover, the presence of MP nucleic acid in the cerebrospinal fluid of both children indicated that the in situ synthesis of MP antibodies or the integrity of the brain-blood barrier was compromised.^[22,23]^ Vasculitis caused by direct invasion of focal MP may also lead to the formation of small embolisms.^[13,19]^

Patients 2 and 3 underwent fiberbronchoscopy under basic anesthesia and developed hemiplegia 2 hours and 1 day after surgery, respectively. The level of D-dimer in children with severe MPP was higher than that in children with mild pneumonia.^[15]^ Inflammation and surgery can trigger an increase in D-dimer levels. Elevated D-dimer levels indicate a severe hypercoagulable state, excessive inflammatory response, and prolonged vascular endothelial injury.^[14]^ Hypoperfusion induced by anesthesia is the main cause of cerebral watershed infarction in adults. The timing of cerebral infarction in these 2 cases clearly suggested that it was related to the operation. Preoperative anesthesia and fasting may accelerate the occurrence of cerebral infarction.

The previous cases of cerebral infarction in the acute stage of MP infection were reviewed, and the cases with head imaging published at the same time were selected. Compared with non-watershed cerebral infarction (10 cases in total)^[24–33]^ (Table 3) and cerebral watershed infarction (A total of 7 children were included,including 4 in this study) group^[34–36]^ (Table 2), coincidentally, 3 of the children with watershed infarction had both other arterial embolism and MP-positive CSF, while none of the 10 children with non-watershed infarction did. The age of 5 years old was the most common age (6/17) for cerebral infarction in acute stage of MP (Tables 2 and 3), especially in children with cerebral watershed infarction group (4/7). For the cerebral watershed infarction group, the children were younger (5 years vs 7.8 years), the mean interval between the onset of respiratory symptoms and the development of stroke was longer (11.4 days vs 7.9 days) (Tables 2 and 3).

**Table 2:** Clinical characteristics and examination findings of MP children in watershed cerebral infarction group.

Reference/Age(y)/Gender	Time of onset of neurological symptoms/Respiratory illness	Neurological symptoms	Cerebral infarction	Other site embolism/triggers	Characteristics of cerebrospinal fluid
Case 1 5/boy	13 d/lung consolidation, pleural effusion	Rrritability, reduced activity of the left upper limb.	Thalamic lacunar cerebral infarction and bilateral cerebellar watershed infarction. Vascular wall thickness is uneven.	Right atrial and left brachial artery thrombosis	The white blood cell count in the CSF was 8 × 10^6^/L.The PCR of the CSF was positive for MP.
Case 2 5/boy	10 d/lung consolidation, pleural effusion	Left hemiplegia.	Posterior cerebral watershed infarcts were detected in the right middle cerebral artery and posterior cerebral artery. Subcortical watershed infarcts were detected in the right half oval center.	The patient developed hemiplegia 2 h after fibreoptic bronchoscopy.	The leukocyte count in CSF was 16 × 10^6^/L. The PCR of the CSF was negative for MP.
Case 3 4/girl	19 d/lung consolidation	Left hemiplegia.	Subcortical watershed infarction at the junction of the middle cerebral artery with the anterior cerebral artery and the posterior cerebral artery.	The patient developed hemiplegia the day after undergoing the second fibreoptic bronchoscopy.	Not assessed
Case 4 7/girl	13 d/lung consolidation	Right hemiplegia	Left Heubner recurrent artery and lenticular artery subcortical watershed infarct. Vascular wall thickness is uneven.	Left tibial artery and bilateral peroneal artery thrombosis.	The leukocyte count was 5 × 10^6^/L in the CSF. The PCR of the CSF was positive for MP.
Ding et al/5/girl^[25]^	10 d/lung consolidation, pleural effusion	Dysarthria, dysphagia, and left hemiplegia.	Lacunar infarction of the midbrain and thalamus and watershed cerebral infarction of the cerebellar.	Right ventricle and left common femoral vein thrombus.	The leukocyte count in CSF was 16 × 10^6^/ L. The PCR of the CSF was positive for MP.
Choi et al 5/boy^[26]^	6 d/lung consolidation	Drowsy, and exhibited dysarthria, limited eye movement, irritability and seizure.	Watershed cerebral infarction of pontus, thalamus and cerebellum.	No	The leukocyte count in CSF was 0 × 10^6^/ L. The PCR of the CSF was negative for MP.
Lee at al 4/boy^[27]^	9 d/lung consolidation, pleural effusion	Irritability and disoriented speech.	Right posterior cortical watershed cerebral infarction.	No	The leukocyte count in CSF was 0 × 10^6^/ L. The PCR of the CSF was negative for MP.

CSF = cerebrospinal fluid, MP = *Mycoplasma pneumonia*, PCR = polymerase chain reaction.

**Table 3:** Clinical characteristics and examination findings of MP children in non-watershed cerebral infarction group.

Reference/Age(y)/Sex	Time of onset of neurological symptoms/Respiratory illness	Neurological symptoms	Cerebral infarction	Other site embolism/triggers	Characteristics of cerebrospinal fluid
Fu et al/5/girl^[15]^	10 d/lung consolidation	Right limb immobility and became aphasic	Infarction of the left lentiform nucleus and external capsule. Occlusion of the left middle cerebral artery.	No	The CSF was normal.
Kong et al/11/girl^[16]^	6 d/ lung consolidation	Right central facial paralysis, right limb immobility.	Left basal ganglia, frontal, temporal, parietal and right temporal lobe, and abnormal signal areas in the lobe, radial corona, and temporal-parietal subcortical regions. Occlusion of the bilateral middle cerebral artery, mainly on the left side.	Diarrhea	The CSF was normal.
Jin et al/7/boy^[17]^	6 d/lung consolidation, plastic bronchitis	The muscle strength of right limb was grade 1, and left limb was grade 3.	Abnormal signal areas in the left frontoinsular cortex, involving the internal capsule and basal Ganglia. Left middle cerebral artery occlusion.	Fibreoptic bronchoscopy	No CSF was checked.
Kang et al/5/girl^[18]^	6 d/pneumonic infiltration and pleural effusion	Left hemiparesis and left facial palsy.	Occlusion of the right middle cerebral artery in the M1 segment.	No	The leukocyte count in CSF was 3 × 10^6^/L. The PCR of the CSF was negative for MP.
Bao et al/8/boy^[19]^	13 d/lung consolidation, pleural effusion	Cried suddenly, shook his right upper limb and blurred vision.	Abnormal signal of the left lentiform nucleus, caudate nucleus and within the temporal lobe.	No	The analysis of CSF showed a leukocyte count of 40 × 10^6^/L. The PCR of the CSF was negative for MP.
Carcia et al/6/boy^[20]^	2 d/Upper respiratory tract infection	Visual impairment and headache.	Cranial CT showing subacute infarction of left occipital lobe and acute infarction in right occipital lobe. CTA revealed marked stenosis of both PCAs.	No	No CSF was checked.
Kim at al/3/girl^[21]^	7 d/pneumonia	Left central facial palsy, motor strength was decreased to grade III/VI in the left arm and leg.	MRI showed an acute infarction in the territory of the right lenticulostriate artery. MRA showed no luminal narrowing or obstruction of regional vessels.	No	The analysis of CSF showed a leukocyte count of 13 × 10^6^/L.
Carcia et al/13/girl^[22]^	5 d/multiple bilateral lung consolidations	Mild left-sided weakness and ataxia.	Brain CT demonstrated multiple bilateral ischemic strokes, in the distribution of the right ACA/MCA and left MCA with right to left midline shift, subfalcine and uncal downward herniation, and global cerebral hypoperfusion.	No	No CSF was checked.
Visudhiphan et al/12/girl^[23]^	10 d/pneumonia	Right limb immobility, right central facial weakness and became aphasic.	Brain CT disclosed a large, low-attenuated area involving both gray and white matter of the left fronto-temporoparietal area and the left basal ganglia causing 1.2 cm of midline shift with tlansfalcine herniation	No	The leukocyte count in CSF was 2 × 10^6^/L.
Antachopoulos et al/8/boy^[24]^	14 d/pneumonia	Muscle strength was diminished in the right arm and leg.	MRI revealed an infarct in the left thalamus also affecting the posterior part of the internal capsule. MRA demonstrated marked stenosis (almost complete occlusion) of the left posterior cerebral artery near the bifurcation of the basilar artery, with faint signal of the distal branches of the left posterior cerebral artery.	No	No CSF was checked.

CSF = cerebrospinal fluid, CT = computed tomography, CTA = computed tomography angiography, MP = mycoplasma pneumonia, MRA = magnetic resonance angiography, MRI = magnetic resonance imaging, PCR = polymerase chain reaction.

At present, the diagnostic criteria for MPP are as follows: typical clinical symptoms and imaging; a single serum anti-MP (particle agglutination test) 1:80, a 4-fold increase or decrease in anti-MP (particle agglutination test) titer between the 2 acute and recovery stages; positive MP PCR results.^[37–40]^

There are several limitations in the study. Autoantibodies, including anti-cardiolipin antibodies and antiphospholipid antibodies, which may be the cause of vasculitis caused by MP, were not tested in all 4 patients. Also, because of the absence of IgE as a result, the relationship between the occurrence of cerebral infarction and IgE is unclear. In addition, 4 children were not treated with drugs such as tetracycline when azithromycin was ineffective, so whether the occurrence of cerebral infarction is related to azithromycin resistance still needs further prospective study.

## 4. Conclusion

In summary, focal vasculitis, hypercoagulability and hypoperfusion may be the dominant causes of watershed cerebral infarction in patients with MP infection.

## Author contributions

**Conceptualization:** Yuan-Yuan Wang, Jun-Ling Cui.

**Data curation:** Jun-Ling Cui, Ge-Fei Li.

**Formal analysis:** Feng Mo.

**Funding acquisition:** Jun-Ling Cui.

**Investigation:** Feng Mo, Ge-Fei Li.

**Supervision:** Yuan-Yuan Wang, Jun-Ling Cui.

**Writing – original draft:** Yuan-Yuan Wang.

**Writing – review & editing:** Jun-Ling Cui.
